# Productivity and Post-Harvest Fungal Resistance of Hot Pepper as Affected by Potassium Silicate, Clove Extract Foliar Spray and Nitrogen Application

**DOI:** 10.3390/plants10040662

**Published:** 2021-03-30

**Authors:** Shimaa M. Hassan, Ahmed F. El-Bebany, Mohamed Z. M. Salem, Doaa A. Komeil

**Affiliations:** 1 Department of Vegetable crops, Faculty of Agriculture (El-Shatby), Alexandria University, Alexandria 21545, Egypt; shaymaa.hassan@alexu.edu.eg; 2 Department of Plant Pathology, Faculty of Agriculture (El-Shatby), Alexandria University, Alexandria 21545, Egypt; ahmed.elbebany@alexu.edu.eg; 3 Forestry and Wood Technology Department, Faculty of Agriculture (El-Shatby), Alexandria University, Alexandria 21545, Egypt; mohamed-salem@alexu.edu.eg

**Keywords:** hot pepper, clove water extract, nitrogen, silica, fungal infection, post-harvest, HPLC analysis

## Abstract

In the present study, growth and productivity of hot pepper planted in the two successive summer seasons of 2017 and 2018 were evaluated under the effect of foliar spray of variable doses of potassium silicate (PS), and clove water extract (CWE) with different rates of nitrogen (N) fertilization application. The post-harvest resistance of hot pepper fruits to *Alternaria alternata* fungal infection, was also evaluated. Maximum plant height was achieved with the application of the highest rates of N, PS and CWE, while the intermediate rates were sufficient to reach the maximum number of branches, the highest leaf dry matter and chlorophyll accumulation. Fruit yield progressively increased with increasing the applied N rate. The foliar application of PS and CWE exerted a limited, yet positive effect on fruit yield. Generally, the least amount of fruit yield, amounting to 18.84 and 18.00 t ha^−1^, resulted from the application of the lowest N rate (144 kg ha^−1^) in the absence of PS and CWE. The highest significant fruit yield, amounting to 31.71 and 31.22 t ha^−1^, for 2017 and 2018, respectively, accompanied the application of the maximum levels of the three factors. The application of high N rates increased the post-harvest *Alternaria* fruit rot severity. The positive effect of CWE application in counterbalancing the negative effects associated with the high rates of N and PS may be related to the presence of phenolic and flavonoid compounds ellagic acid, benzoic acid, catechol gallic acid, rutin, myricetin, quercetin, apigenin and kaempferol as identified by High Performance Liquid Chromatography (HPLC).

## 1. Introduction

Hot pepper (*Capsicum annuum)*, a member of the Solanaceae family, is a high cash-value vegetable crop worldwide [1], for both fresh market and culinary purposes. Hot pepper, originated in South America, is cultivated in temperate and warm climates and is valued for its characteristic pungency, aroma and color appeal. The *Capsicum* genus includes different plants like, red pepper, chili pepper, Tabasco pepper, African chilies, cayenne pepper, paprika and Christmas pepper [2]. These are important commercial spice and vegetable crops for small and marginal farmers in Asia, Africa and South America. Egypt is the second largest pepper producer in Africa, after Nigeria [3]. Pepper thrives in environments with rising seasonal temperatures, in other words, between 18 and 27 °C during the day and between 15 and 18 °C at night [1].

The nutritional elements received by the capsicums affect crop performance, its resistance to pests and diseases, and, thus, reflect on crop productivity. To overcome these stresses, vegetables require an optimum supply of macro and micronutrients. Nitrogen (N) fertilizer is a key component for pepper growth and development, and is often a limiting factor for high productivities [4]. Nonetheless, controversial results were reported concerning the suitable amounts of N fertilizer needed to reach the optimum productivity for pepper cultivation. While, Khan et al. [5] reported a significant offset in pepper productivity that accompanied the application of high N rates. Nonetheless, the application of high N rates resulted in rising costs of the agricultural practice, thus proving uneconomic, in addition to its consequent adverse environmental implications [6].Therefore, there is a crucial need to determine the proper amount of N fertilizer that would increase the productivity and quality of pepper crops, while reducing the costs and harmful effects on the environment. In this regard, smart management practices are recently receiving increased attention, to reduce the amounts of applied chemical fertilizers in vegetable cropping systems. The use of silicon-based (Si) fertilizers has been reported to improve N uptake. It is evident that some field crops (e.g., sugarcane, rice, wheat and maize) can absorb Si quickly and in great quantities [7].In recent years, the number of studies reporting the effects of Si application to crops has increased substantially, especially in vegetable crops such as pea [8], cowpea [9], and cucumber [10]. This increased interest in Si is likely due to its beneficial effects on plant resistance to abiotic and biotic stresses such as insects and pathogens [11], salt and drought stresses [12], and heavy rain and wind [13]. Silicon has also been reported to improve crop yield [14], plant growth, plant architecture, erectness, and photosynthesis rate [15], to decrease transpiration rate [16], and to reduce water use [14].

Capsicum peppers are in high demand in the international and national markets. The fresh fruit market generally prefers a healthy fruit, free of spoilage with extended shelf-life of not only a few days. Chemical control remains the main measure to reduce the incidence of postharvest diseases in various fruits and vegetables. Meanwhile, the continuous use of fungicides is not only expensive but also hazardous for all living organisms. Thus, following alternative economic, yet environmentally-friendly methods is highly encouraged [17]. Among the fungal diseases affecting pepper, *Alternaria alternata* (Fries) Keissler is one of the most economically important; it is responsible for both pre- and post- harvest crop losses, and consequently affects both yield and quality. The black mold infection can be developed during transport and storage of fresh pepper fruit [18,19]. The distinct fruit rot can cause substantial economic losses, since diseased fruits become unmarketable.

Extracts from clove (*Syzygium aromaticum* L., Myrtaceae family) are reported as a potential bio-fungicide [17]. Several phytochemical compounds such as phenolic and flavonoid; gallic acid, syringic acid, protocatechuic acid, caffeic acid, eugenin, eugenitin, P-hydroxybenzoic acid, salicyclic acid, kaempferol, quercetin, rhamnetin, phenylacetic acid, myricetin and isohamnetin were isolated and identified in clove bud extract [18]. The extract exhibited bioactivities against plant pathogenic fungi, *Fusarium oxysporum, Rhizoctonia solani*, and *Alternaria solani* and against *F. oxysporum, Aspergillus niger, Penicillium* sp. and *Trichoderma* sp. [20], where gallic acid, ellagic acid, quercetin, kaempherol, myricetin, and apigenin were isolated from the extract. Furthermore, clove essential oil with its main compound, eugenol, was responsible for its antifungal properties [21]. Clove oil was reported to be effective at suppressing *R. solani* and *F. oxysporum* [22].

Capsicum peppers are in high demand in the international and national markets. The fresh fruit market generally prefers a healthy fruit, free of spoilage with extended shelf-life of not only a few days. Chemical control remains the main measure to reduce the incidence of postharvest diseases in various fruits and vegetables. Meanwhile, the continuous use of fungicides is not only expensive but also hazardous for all living organisms. Thus, following alternative economic, yet environmentally-friendly methods is highly encouraged [23]. Among the fungal diseases affecting pepper, *Alternaria alternata* (Fries) Keissler is one of the most economically important; it is responsible for both pre- and postharvest crop losses, and consequently affects both yield and quality. The black mold infection can be developed during transport and storage of fresh pepper fruit [24,25]. The distinct fruit rot can cause substantial economic losses, since diseased fruits become unmarketable.

It was hypothesized that in presence of potassium silicate and clove extract, lower amounts of nitrogen fertilizer might be needed to reach the maximum fruit yield from hot pepper plants. In addition, using clove extract would increase the post-harvest shelf-life of pepper fruits by preventing *A. alternata* infection, or at least reducing its severity. Objectives of the current study were thus, evaluating some vegetative and fruit characteristics, and fruit macronutrients’ contents, as affected by the application of nitrogen fertilization, potassium silicate, and clove water extract, each applied at three different levels. In addition to testing the effects of the three factors and their interactions on the severity of *A. alternata* fruit infection as an indication to the postharvest shelf-life of pepper fruits.

## 2. Results

### 2.1. Phenolic and Flavonoid Compounds in Clove Water Extract

**Figure 1:**
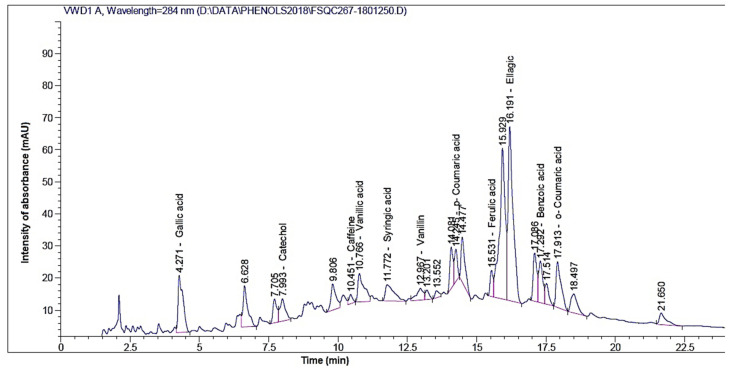
High Performance Liquid Chromatography (HPLC) chromatogram of phenolic compounds present in clove extract.

**Figure 2:**
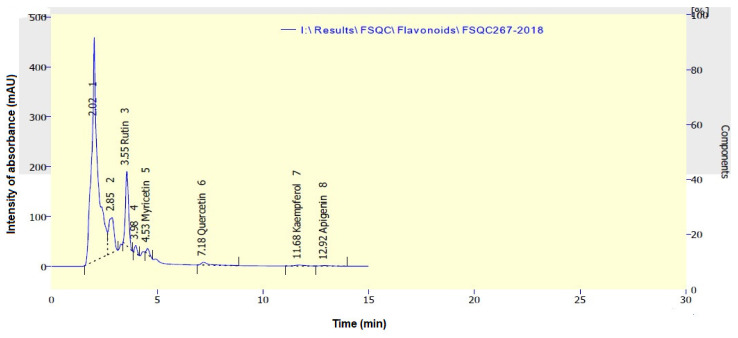
HPLC chromatogram of flavonoid compounds present in clove water extract.

The identification of phenolic and flavonoid compounds in clove water extract (CWE) are shown in Figure 1 and Figure 2, respectively. The highest amounts of phenolic compounds (Table 1) were ellagic acid (100.83 mg/100 g extract), benzoic acid (106.22 mg/100 g extract), catechol (20.15 mg/100 g extract), gallic acid (13.26 mg/100 g extract), and vanillic acid (11.39 mg/100 g extract); while rutin (7588.01 mg/100 g extract), myricetin (77.71 mg/100 g extract), quercetin (68.32 mg/100 g extract), apigenin (30.35 mg/100 g extract), and kaempferol (29.71 mg/100 g extract) were presented as identified flavonoid compounds in CWE (Table 1).

### 2.2. Field Experimental Results

For all studied parameters, main effects of the tested factors will be presented and discussed only if the interactions involving them are not significant.

#### 2.2.1. Vegetative Characteristics

Analysis of variance (ANOVA) revealed that, plant height, number of branches, and leaf dry matter (DM) content were highly significantly variables (*p* < 0.001) as affected by the tested nitrogen fertilizer (N) applications, potassium silicate (PS), and clove water extract (CWE) foliar spray, as main effects. Interactions between the studied factors were non-significant for the three parameters (*p* > 0.05).

Means presented in Table 2 demonstrated a progressive increase in plant height with increasing N rate, the significantly tallest plants were attained with the application of 288 kg N ha^−1^, which represented an increase of 14.76 and 14.12%, for 2017 and 2018, respectively, over the plant height achieved with the application of the lowest N rate (144 kg N ha^−1^). Meanwhile, the maximum number of branches per plant was a result of the application of the intermediate N rate, amounting to 6.74 and 6.81 for 2017 and 2018, respectively, while the least number of branches was a result of the lowest N rate. Similarly, the application of 216 kg N ha^−1^ resulted in the highest leaf DM accumulation (35.27 g kg^−1^) in 2017, while in 2018 a non-significant difference among the three tested N rates was observed.

Concerning the significant effect of the PS treatments on the plant vegetative characteristics, it was observed from Table 2 that the tallest plants were achieved with the application of 250 and 500 PS mg L^−1^, during both seasons. On the other hand, the intermediate PS level was superior to the other two treatments in the production of highest significant number of branches per plant. The highest significant leaf DM accumulation was achieved with the highest level of PS amounting to 35.59 and 35.45 g kg^−1^, during 2017 and 2018, respectively. Observably, the tallest significant plants were achieved with the application of 10,000 mg L^−1^ CWE during 2017 and the application of 5000 and 10,000 mg L^−1^, during 2018. Moreover, spraying the plants with the intermediate CWE level resulted in the production of the highest significant number of branches per plant. Both the highest and intermediate CWE levels resulted in the highest significant leaf DM accumulation compared to the control treatment.

Leaf chlorophyll content was significantly affected by the N × PS interaction during both seasons (Table 3). When 144 kg N ha^−1^ was applied, non-significant effect of the three tested PS levels was detected during both seasons. Nonetheless, at the intermediate N rate, the application of 0 or 250 mg PS l^−1^ resulted in the highest significant chlorophyll content, during both seasons, compared to the highest PS level. On the contrary, the intermediate and highest PS levels (250 and 500 mg L^−1^) were superior to the control treatment, during both seasons, when accompanied with the application of the highest N rate. Moreover, at 0 and 250 mg PS L^−1^, the intermediate N rate (216 kg ha^−1^) was sufficient to achieve the highest significant leaf chlorophyll content during both seasons, while when increasing the PS level to 500 mg L^−1^, the three tested N rates were non significantly different during 2017. On the other hand, during 2018, the application of 216 kg N ha^−1^ was inferior to the other two treatments when a level of 500 mg PS L^−1^ was sprayed.

#### 2.2.2. Fruit Characteristics and Yield

Results of the ANOVA revealed that the fruit diameter was significantly variable only as affected by the sprayed CWE levels (*p* < 0.01) during 2017 and 2018, while fruit length was significantly affected by the two-way interactions; N × PS and N × CWE (*p* < 0.001). The three-way interaction N × PS × CWE was only significant in the case of fruit yield during both seasons.

As illustrated in Table 4, little, yet significant, variation was detected in the fruit diameter as affected by the tested CWE levels during both seasons. The application of 5000 mg CWE L^−1^ resulted in 0.07- and 0.11-mm increase in the fruit diameter for 2017 and 2018, respectively. Increasing the CWE level to 10,000 mg L^−1^, increased the fruit diameter by 0.08 and 0.15 mm, for the two respective seasons. For all tested PS levels, the highest N rate resulted in the significantly tallest fruits, reaching an average of 13.58 and 13.63 cm for 2017 and 2018, respectively. A less pronounced variation in fruit length was detected with varying PS applications. On the other hand, a clearer variation was detected in the fruit length with varying CWE applications as affected by the N × CWE interaction (Table 5). It was clear that the highest CWE level significantly increased fruit length especially at lower N rates, while at higher N rates non-significant variation among CWE levels was detected.

Fruit yield was significantly variable as affected by the N × PS × CWE interaction during both seasons. Observing the fruit yield means presented in Table 6, revealed that increasing the N rate, across all tested PS and CWE levels, led to significantly increasing the fruit yield. Similarly, spraying the plants with CWE, either 5000 or 10,000 mg L^−1^, increased the fruit yield compared to the control treatment. A positive effect was also reported for the application of PS on the fruit yield as compared to the control. Despite the consistent direction of variation in response to the three tested factors, variable magnitude of variation was reported for the three factors, which might have contributed to the significant three-way interaction. Hence, the greatest variation in fruit yield accompanied the application of different N rates, while the variation resulting from the application of PS and CWE was limited. In general, the lowest fruit yield, amounting to 18.84 and 18.00 t ha^−1^, resulted from the application of the 144 N kg ha^−1^ in the absence of PS and CWE. However, spraying with 250 mg PS L^−1^ and 5000 mg CWE L^−1^, with 144 kg N ha^−1^, increased the yield by 16.77 and 20.77% during 2017 and 2018, respectively. A similar trend was observed for other treatment combinations. On the other hand, the highest significant fruit yield, amounting to 31.71 and 31.22 t ha^−1^, for 2017 and 2018, respectively, accompanied the application of the maximum levels of the three factors. These values represented an increase of 31.03 and 28.42%, for 2017 and 2018, respectively, over the yield values resulting from the application of 288 kg N ha^−1^ alone.

#### 2.2.3. Severity of Post-Harvest *Alternaria alternata* Infection

Post-harvest disease severity of *A. alternata* was assessed 10 and 15 days after infection (DAI) in terms of measuring the diameter (mm) of the rot lesion that appeared on pepper fruits. ANOVA revealed that this parameter was significantly affected by the N × PS × CWE interaction. When measured at 10 DAI, means presented in Table 7 demonstrated increased infection severity of pepper fruits after treatment with the highest N rate (288 kg ha^−1^) across all PS and CWE levels. Nonetheless, spraying the pepper plants with CWE significantly decreased the *A. alternata*, infection severity, while, a less pronounced effect was observed for the PS treatments. In general, the highest significant infection severity was reported for the application of 288 kg N ha^−1^ + 250 mg PS L^−1^ at zero CWE. On the other hand, the least significant infection severity accompanied the application of 288 kg N ha^−1^ + 500 mg PS L^−1^ + 10,000 mg CWE L^−1^. This result highlighted the positive effect of CWE application in counterbalancing the negative effects associated with the high rates of N and PS. Noticeably, for all treatment combinations, the pepper fruits at 15 DAI showed an increased infection severity compared to 10 DAI, however, the magnitude of increase was variable among the different treatment combinations. Again, increasing the rate of applied N was accompanied with an increase in the severity of infection measured at 15 DAI, which was counterbalanced by the addition of 10,000 mg CWE L^−1^ in presence of 0 or 250 mg PS L^−1^. On the contrary, at 500 mg PS L^−1^, the infection severity decreased by increasing the N rate, with the highest values accompanying the application of 144 kg N ha^−1^, while the positive effect of CWE spraying on decreasing the infection severity remained constant. When measured at 15 DAI, the highest significant infection severity was achieved in the case of application of 216 or 288 kg N ha^−1^ + 250 mg PS L^−1^ with zero CWE.

## 3. Discussion

### 3.1. Nitrogen Fertilizer-Related Effects

Nutrient management is a very important agricultural practice that needs to be precisely adjusted to achieve optimum yield with satisfactory nutritional value from *Capsicum* spp. [26]. Due to the limited initial soil nutrients’ composition of the experimental site, the pepper plants were expected to greatly benefit from the applied nutrients in both the vegetative growth and fruit developmental stages. This assumption was confirmed with the limited vegetative growth and inferior fruit characteristics observed, in the current study, with the lowest N rate in absence of PS and CWE applications. On the other hand, a significant development in all tested parameters was detected with the advanced rates of the three tested nutrients.

Among the tested nutrients in the current study, N fertilizer rates exerted the most pronounced effect on the vegetative characteristics of hot pepper plants. Nitrogen fertilization stimulates plant growth [27], and plant height is a very important determinant to the plant’s vigor and, thus, to its growth and productivity [26]. In the current study, a progressive increase in the plant height was observed by increasing the applied N rate. This result was in agreement with the findings of several researchers, for example, [27,28], and could be attributed to the extensive increase in cell growth rate with higher N rates, through stimulating the meristematic activity and thus producing more tissues and organs [29] resulting in taller plants. On the other hand, the application of the highest N rate might encourage stem lodging and, thus, negatively impact the final yield [30], thus this is not fully recommended. Unlike the plant height, the application of the intermediate N rate (216 kg N ha^−1^) was sufficient to reach the highest number of branches per plant and leaf DM content. Thus, proper vegetative growth attributes could be reached with only 216 kg N ha^−1^, while avoiding the risk of lodging and the negative environmental implications accompanying the higher N levels. In accordance with the current study, Ayodele et al. [31] reported that increasing N fertilizer application enhanced the dry matter yield partitioning into root, stem, and leaves for hot pepper plants. Nonetheless, application of adequate N rates increases the plant’s chlorophyll content resulting in dark green coloration, as N improves the plant’s ability to turn solar energy into sugars, which is essential for growth and, thus, accumulation of photosynthates and chlorophyll formation. In addition, the increase in protein content of the whole plant with N fertilization, might be reflected in better vegetative growth with abundant chlorophyll content, owing to the participation of protein in the development of all plant parts including chlorophyll disposition [31].

The detected positive effect of N fertilizer application of the hot pepper plant’s vegetative growth was reflected in the fruit yield, its characteristics, and chemical composition. Similar to the current findings, Aminifard et al. [26] and Aminifard and Bayat [28], reported a significant increase in sweet peppers’ fruit yield by increasing N fertilization rate, which they attributed to the stimulating effect of N on the plant’s vegetative characteristics, consequently, flower and fruit formation. Similar positive effects of N fertilization on fruit yields of hot peppers and habanero peppers were reported by Ayodele et al. [31], and Medina-Lara et al. [32], respectively. The increased fruit yield with N application would be attributed to several interacting factors during plant growth, for example, the presence of adequate N supply would boost plant growth and reduce competition for soil nutrients. In addition, higher N supply would increase the number and size of fruits which would result in higher fruit yield [33]. Moreover, the presence of abundant N supply stimulates the flowering process, thus, more flowers are formed resulting in higher fruit set and consequently increased fruit yield [32]. Similar results were reported by Aujla et al. [34], who recommended the application of increasing N rates for better weight and volume of pepper fruits. Nonetheless, Shakouri et al. [35] achieved highest fruit yield and fruit characteristics from green pepper with the application of the highest N rate (240 kg N ha^−1^).

### 3.2. Potassium Silicate-Related Effects

Potassium silicate is a source of highly soluble potassium and silicon. It is applied in agricultural production systems primarily as a silica amendment, and has the extra advantage of supplying small amounts of potassium. Silicon is the second most abundant element within the earth’s crust. Generally, silicon has not been considered an important plant nutrient in the past, despite its proven beneficial effects on plant growth and disease prevention [36,37,38]. In the current study, a linear positive effect of PS addition on plant height of hot pepper plants was reported. Meanwhile, the intermediate PS level (250 mg L^−1^) was sufficient to reach the highest significant number of branches with the maximum leaf area. In similar environmental conditions to the current study, Abdelaziz and Geeth [39] reported the positive effect of silicon foliar spray on growth and vegetative characteristics of sweet pepper plants. They attributed this positive effect to the silicon deposition in the cell wall of xylem vessels, which prevents the compression of the vessels under the condition of high transpiration caused by high summer temperatures during the growing season [40]. In addition, the accumulation of foliar sprayed silicon on the leaf surface produces a thick silicate layer that will effectively reduce the transpiration rate by around 30%. Therefore, silicon is considered an antitranspirant and recommended as foliar spray in hot and dry environments, like the current study. In their study on the effect of silicon application on chili pepper plants, Sudradjat et al. [41] reported a significant increase in leaf length with silicon application, while similar to the current study, Pereira et al. [42] concluded that silicon application increased the leaf area, which was significantly reflected in higher photosynthesis rates, thus, uplifting the plant’s vegetative growth. The improved photosynthesis with the application of PS, in the current study, was probably the main driving force behind the increased chlorophyll content. Similar observations was reported by Kamal [43] for sweet pepper plants. Nonetheless, PS containing a considerable amount of K_2_O is reported to enhance the total dry mass accumulation of sweet pepper plants, which was the case in the current study with the application of PS increasing the DM accumulation of the plant. This observation might be attributed to stomatal regulation by potassium and the corresponding higher rates of photosynthesis. Moreover, potassium is considered to be a key promoter for the growth of meristematic tissues, which, in presence of high N rates would significantly boost the vegetative growth [39].

Limited, yet significant effect was observed in the current study for PS application on hot pepper fruit yield. Fruit yield increased with increasing the PS dose, yet with a small magnitude. The positive effect of PS was most probably attributed to its ability to improve flowering, fruit set percentage, and fruit weight. In addition, silicon is reported to increase the thickness and hardiness of the pepper fruits as it accumulates in the epidermal tissues, improving the plants pest resistance on the one hand and strengthening the plants and fruits on the other hand [39]. In accordance with the current study, an investigation conducted in India reported that the foliar application of 0.4% silicon (as potassium silicate containing 18% silicon) resulted in the highest significant chili pepper fruit yield [44].

### 3.3. Clove Extract-Related Effects

Clove water extract (CWE) showed the presence of some important phenolic and flavonoid compounds including ellagic acid, benzoic acid, catechol, gallic acid, vanillic acid, rutin, myricetin, quercetin, apigenin, and kaempferol. Previously, gallic acid was found in high amounts (2375.8 mg/100 g) and other phenolic acids (caffeic, ferulic, ellagic, and salicylic) and flavonoids such as kaempferol and quercetin were also found [45]. Amounts of mg/100 g of gallic acid (847.36), syringic acid (259.04), protocatechuic acid (252.29), caffeic acid (151.01), eugenin (121.30), eugenitin (101.29), P-hydroxybenzoic acid (85.04), salicyclic acid (31.84), kaempferol (30.75), quercetin (27.68), rhamnetin (21.61), phenylacetic acid (18.09), myricetin (16.67), and isohamnetin (5.07) were identified in the methanol extract of clove buds [18]. *S. aromaticum* extract exhibited moderate antifungal activities against plant pathogenic fungi, *Fusarium oxysporum*, *Rhizoctonia solani*, and *Alternaria solani* [19]. Gallic acid, gallic acid 3-O-β-D-(6′-O-galloyl)-glucopyranoside, ellagic acid, myricetin, quercetin, kaempherol, and apigenin were isolated from the methanolic extract of clove flower buds, which observed strong inhibitory effect against *F. oxysporum*, *Aspergillus niger*, *Penicillium* sp., and *Trichoderma* sp. [23]. CWE at 20% showed 100% inhibition of *Aspergillus niger* mycelial growth [46]. Furthermore, extracts *S. aromaticum* were found to be potent against the growth of *Aspergillus* spp. and *Penicillium* spp. [47,48].

In the present study, spraying the hot pepper seedlings with clove extract caused a significant improvement to the studied vegetative growth characteristics. Maximum plant height was achieved with the foliar application of the highest CWE dose, which might be attributed to the increased cell division and cell enlargement. The intermediate CWE dose was sufficient to reach the maximum number of branches with maximum leaf dry matter content from hot pepper. Fruit characteristics and yield were little, yet positively affected by the foliar application of CWE. It is evident that the foliar application of several types of extracts would exert a bio-stimulation effect on the growth and development of several plant species, for example, tomato [49], *Origanum majorana* [50], and geranium plants [51]. This might be attributed to the promoted biomass production as a result of enhancing the shoot and root fresh weights. The effect of the CWE on enhancing plant growth of pepper was quite similar to the effect of other organic compounds such as humic acids, amino acids, salicylic acid and vitamins [52,53,54,55]. Nonetheless, the foliar application of essential oils to several vegetable crop species was known to improve the nutrient uptake and nutrient use efficiency, thus resulting in a better growth, in this regard, the effect of the extract was similar to the humic substances [49,54].

### 3.4. Severity of Post-Harvest Alternaria alternata Infection

Resistance to infection by post-harvest diseases is an essential factor for the economic value of the agricultural commodity [56]. Pre-harvest practices such as plant balanced-fertilization and spraying of plant defense activation agents are known to increase disease resistance. Accumulation of phenolic compounds in plants is well known as a disease resistance mechanism [57]. The current investigation revealed that pre-harvest spraying of hot pepper by clove extract improved the resistance of pepper fruit to the fungal pathogen *A. alternata*.

CWE is recognized for its antioxidant and antimicrobial effects [21], with especial emphasis on its antifungal activities against several fungal species, for example, *A. alternata*, *Fusarium chlamydosporum*, *Helminthosporum oryzae*, and *Rhizoctonia bataticola* [58]. In their investigation, Thabet and Khalifa [59] reported strong antifungal activities of clove extract against several fungal pathogens of tomato. In the current study, the resistance of hot pepper fruit to *A. alternata* could be as a result of phenolic compound constituents of the CWE as it contains significant concentrations of catechol, p-coumaric, and ferulic acids. It has been reported that accumulation of caffeic, ferulic acids, and rutin in another solanaceous plant, namely, potato, enhanced resistance to the fungal pathogen *Verticillium dahliae* [57]. Similar values of effective application of silicon were reported by Rodrigues [60], who recommended the application of silicon through leaves, which only accumulates on the canopy, leaves, and stem. Silicon provided resistance to disease but it did not inhibit the development of pathogens directly. In addition, Si acts on the modulation of gene expression and signaling through phytohormones, decreasing the effectivity of ethylene levels, which delay the senescence under dry conditions [61].

## 4. Materials and Methods

### 4.1. Site Description

Greenhouse experiments were carried out during two successive summer seasons (2017 and 2018) at the Experimental Station of the Faculty of Agriculture, Alexandria University, in Alexandria, Egypt. The monthly weather under greenhouse experiments are shown in Table 8. Before cultivating, one-time soil samples were collected at 20–30 cm depth, and analyzed at the central laboratory of Faculty of Agriculture, according to Page et al. [62]. Physical and chemical properties of the experimental soil are presented in Table 9.

### 4.2. Experimental Design and Treatments

Seedlings of hot pepper, cultivar Omega F1, were transplanted in plastic houses on February first, in both winter seasons (2017 and 2018). A split-split-plot experimental design with three replicates, was adopted to study the influence of nitrogen fertilization, the application of different potassium silicate and clove extract concentrations on vegetative parameters, fruit characteristics, and fruit chemical composition of hot pepper plants. Main plots were assigned to test three levels of nitrogen fertilizer, amounting to 144, 216, and 288 kg ha^−1^. The two concentrations of potassium silicate (250 and 500 mg L^−1^) in addition to the control treatment with tap water were tested in the sub-plots. Finally, the sub-sub plots were dedicated to the three concentrations of clove extract (0, 5000, and 10,000 mg L^−1^). Each main plot contained three rows, each row was 20 m long and 1 m wide, making a total main plot area of 60 m^2^.

The levels of nitrogen tested in the present study were 144 (N_1_), 216 (N_2_), and 288 (N_3_) kg N h^−1^. Nitrogen fertilizer was applied in the form of calcium nitrate (15% N), once per week starting from one week after transplanting through drip irrigation system (Table 10), according to [63,64].

Potassium silicate (K_2_SiO_3_), in a powder form containing 22.5% SiO_2_ and 10.25% K_2_O, was applied at two concentrations (250 and 500 mg L^−1^). Plants were sprayed three times, at 30, 45, and 60 days after infection (DAI) along with untreated control (sprayed with tap water) and clove extract were applied as foliar treatments twice, at 40 and 60 DAI.

All the experimental plots received equal amounts of water at equal intervals to ensure homogeneity of irrigation, and avoid any drought stress. Thus, the drip irrigation was scheduled on a regular basis, with the rate of 4 L water per hour. From the second week to the sixth week after transplanting, drips were opened for all plots for 15 min per day, twice a week, thus, each plot received 2 L of water per week during vegetative growth. Starting from the seventh week, drips were opened for all plots for 30 min per day, twice a week, thus, each plot received 4 L of water per week during fruiting growth, on a regular basis.

### 4.3. Preparation of Clove Water Extract

For the preparation of the clove extract, air dried flower buds of *Syzygium aromaticum* were bought from a herbarium store in Alexandria city, Egypt. The material was ground into fine powder using a small laboratory mill and 100 g were extracted by soaking method in 200 mL distilled water (DW) for 3 days, filtered, and concentrated to dryness using a rotary evaporator. The collected extract was stored at 4 °C prior to analysis [65]. The clove water extract was prepared at concentrations of 0.5% and 1%, by dissolving the extract in DW. Clove extract was sprayed twice, at 40 and 80 days after transplanting. The untreated plants (control) were sprayed with tap water plus the same spreading agent only.

### 4.4. Instrument Condition for Phenolic Compounds

Agilent1260 infinity HPLC Series (Agilent, USA), equipped with Quaternary pump, a Zorbax Eclipse plusC18 column 100 mm × 4.6 mm i.d. (Agilent technologies, Santa Clara, CA, USA), was operated at 30 °C. The separation is achieved using a ternary linear elution gradient with (A) HPLC grade water 0.2% H_3_PO_4_ (*v*/*v*), (B) methanol, and (C) acetonitrile. The injected volume was 20 μL. Detection: Variable Wavelength Detector (VWD) set at 284 nm.

### 4.5. Instrument Condition for Flavonoids

HPLC, Smart line, Knauer, Germany, equipped with binary pump, a Zorbax Eclipse plusC18 column 150 mm × 4.6 mm i.d., (Agilent technologies, USA), operated at 35 °C. Eluent: methanol: H_2_O with 0.5% H_3_PO_4_, 50:50 with flow rate 0.7 mL/min, and the injected volume was 20 μL. Detection: UV detector set at 273 nm and data integration by clarityChrom@ software.

### 4.6. Source of Phosphorus

Phosphoric acid (61.5% P_2_O_5_), as a source of phosphorus, was used at the rate of 192 L h^−1^. All the hot pepper fertilization treatments were carried out in accordance with the recommendations of the Ministry of Agriculture and Land Reclamation of Egypt for commercial production. Schedules of fertigation system applied for pepper plants are given in Table 3.

### 4.7. Data Collection

Five pepper plants per plot were randomly sampled at three months after transplanting to determine the vegetative in terms of; plant height (cm) and number of branches per plant. In addition, leaf chemical constituents were determined as total chlorophyll content (mg g^−1^ fresh weight) by using chlorophyll fluorometer (model OPTI-SCIENCES OS-30), Opti-sciences, Inc. USA. Dry matter content (mg/100 g fresh weight) was determined by drying the leaves at 70 °C until constant weight was reached.

Hot pepper fruits at the colored marketable stage were harvested twice weekly along each growing season to determine total yield (t h^−1^). At harvesting, a sample of five randomly chosen fruits of each experimental plot from the second harvest were taken to determine fruit’s physical characteristics in terms of fruit length (cm) and fruit diameter (cm). Samples of hot pepper fruits of the second growing season were subjected to experimental lab to determine decay rate after postharvest infection with *Alternaria alternata.*

### 4.8. Preparation of A. alternata Inoculum and Pathogenicity Test on Pepper Fruit

An identified isolate of *A. alternata* was provided by the Department of Plant Pathology, Faculty of Agriculture, Alexandria University. *A. alternata* isolate was maintained on potato dextrose agar medium for 10 d at 20 °C. The actively grown pathogen culture was used for preparing conidiospore suspension by adding sterile distilled water to the culture plate, and then scraping with a sterile blade to harvest the fungal spores. *A. alternata* spore suspension was adjusted to 10^5^ spores mL^−1^ using a hemocytometer [66].

The resistance of pepper fruits to *A. alternata* was assessed to investigate the effect of the spraying and fertilization treatments on the plant defense system. Physiologically mature pepper fruits that were uniform in size and color, healthy, and free from wounds, were selected for the pathogenicity test. Fruits were washed with tap water and surface sterilized with 70% ethyl alcohol. Water free of conidiospores served as control. Each sterilized fruit was wounded (1.5 mm diameter × 4 mm deep) at two locations using a sterilized pipette tip. Each wound was inoculated with 40 µL of *A. alternata* conidiospore suspension (10^5^ spores mL^−1^) [66]. The inoculated fruits were immediately placed, at room temperature, inside folded-closed polyethylene bags for 10 and 15 d in order to permit symptoms to develop. At the end of the incubation period, the severity of developed symptoms on pepper fruits was scored. Severity was expressed as the area of circle whose diameter was the mean of the length and width of the necrotic area on pepper fruits. The disease severity assessment was repeated at least once with 3 replicates of each treatment.

### 4.9. Statistical Analysis

Data were subjected to analysis of variance (ANOVA) using the SAS software package (SAS Institute, Inc., Cary, NC, USA). Studied parameters (P) and their interactions were statistically analyzed, separately for the two growing seasons, using the following model, with only replicates considered random:

Pijkl=µ+Ri+Nj+eij+PSk+eijk+CWEl+Nj×PSk+Nj×CWEl+PSk×CWEl+Nj×PSk×CWEl+eijkl

where: µ is the overall mean, *R_i_* is the replicate effect (*i* = 1,2,3), *N_j_* is the nitrogen fertilizer treatment effect (*j* = 1,2,3), *e_ij_* is the experimental error “a”, *PS_k_* is the potassium silicate effect (*k* = 1,2,3), *e_ijk_* is the experimental error “b”, CWE*_l_* is the clove water extract effect (*l* = 1,2,3), and *e_ijkl_* is the experimental error “c”. Least significant difference (LSD) procedure at the 0.05 level of probability was used for means’ comparisons.

## 5. Conclusions

The current study highlighted an opportunity for potential improvement in hot pepper growth and productivity, as well as post-harvest resistance to fungal infections, through manipulating nitrogen fertilization and potassium silicate and clove extract foliar spray. The highest significant fruit yield, amounting to 31.47 t ha^−1^, in average of both growing seasons, accompanied the application of the maximum levels of the three factors. The application of high N rates increased the *A. alternata* infection severity in fruits after post-harvest, while, spraying hot pepper plants with CWE significantly decreased the infection severity, highlighting the positive effect of CWE application in counterbalancing the negative effects associated with the nitrogen fertilization and potassium silicate foliar spray. In similar conditions to the current study, fertilizing hot pepper with 216 kg N ha^−1^, accompanied with PS and CWE foliar spray at the rate of 250 mg L^−1^ and 10,000 mg L^−1^, respectively, is recommended.

## Figures and Tables

**Table 1:** Chemical composition analysis of phenolic and flavonoid compounds of clove water extract by HPLC.

Compound	mg/100 g Extract
**Phenolic**	
Gallic acid	13.26
Catechol	20.15
*p*-Hydroxy benzoic acid	ND
Caffeine	2.03
Vanillic acid	11.39
Caffeic acid	ND
Syringic acid	6.39
Vanillin	3.18
*p*-Coumaric acid	2.26
Ferulic acid	3.06
Ellagic acid	100.83
Benzoic acid	106.22
*o*-Coumaric acid	6.48
Salicylic acid	ND
Cinnamic acid	ND
**Flavonoids**	
Rutin	7588.01
Myricetin	77.71
Quercetin	68.32
Naringenin	ND
Kaempferol	29.71
Apigenin	30.35

**Table 2:** Means of plant height (cm), number of branches per plant, and leaf dry matter content (g kg^−1^) as affected by the nitrogen fertilization, potassium silicate and clove water extract main effects during 2017 and 2018.

Treatment		Plant Height		Number of Branches		Leaf DM Content	
		Summer 2017	Summer 2018	Summer 2017	Summer 2018	Summer 2017	Summer 2018
Nitrogen fertilization (kg ha^−1^)	144	70.48 * c	70.77 b	5.44 c	5.03 c	34.45 b	34.25 a
	216	76.92 b	78.37 a	6.74 a	6.81 a	35.27 a	35.16 a
	288	80.88 a	80.63 a	5.81 b	6.11 b	34.06 b	33.85 a
Potassium silicate (mg L^−1^)	0	74.07 b	73.81 b	5.74 b	5.74 b	33.95 b	34.02 b
	250	77.22 a	77.88 a	6.29 a	6.37 a	34.23 b	33.79 b
	500	77.00 a	77.77 a	5.96 ab	5.82 b	35.59 a	35.45 a
Clove water extract (mg L^−1^)	0	74.33 c	74.00 b	5.59 c	5.70 b	33.22 b	33.33 b
	5000	76.18 b	77.07 a	6.40 a	6.22 a	35.09 a	34.80 a
	10000	77.77 a	78.40 a	6.00 b	6.03 ab	35.46 a	35.13 a

* Means followed by different small letter(s) within the same column and studied factor, are significantly different according to the least significant difference (LSD) test at 0.05 level of probability.

**Table 3:** Means of leaf chlorophyll content (g kg^−1^) as affected by the nitrogen fertilization × potassium silicate interaction during 2017 and 2018.

Potassium Silicate (mg L^−1^)	Nitrogen Fertilization (kg ha^−1^)					
	Summer 2017			Summer 2018		
	144	216	288	144	216	288
0	61.26 * aB	64.70 aA	59.27 bC	61.23 aB	65.50 aA	59.41 bC
250	62.91 aAB	64.60 aA	61.31 abB	62.93 aA	65.89 aA	63.05 aA
500	61.93 aA	61.94 bA	62.82 aA	62.66 aA	58.57 bB	62.66 aA

* Means followed by different small letter(s) within the same nitrogen fertilization treatment, and different capital letter(s) within the same potassium silicate treatment, for each growing season, are significantly different according to the LSD test at 0.05 level of probability.

**Table 4:** Means of fruit diameter (mm), and fruit N content (g kg^−1^) as affected by the nitrogen fertilization, potassium silicate, and clove extract’s main effects during 2017 and 2018.

Treatment		Fruit Diameter	
		Summer 2017	Summer 2018
Nitrogen fertilization (kg ha^−1^)	144	1.22 * a	1.20 a
	216	1.25 a	1.24 a
	288	1.28 a	1.27 a
Potassium silicate (mg L^−1^)	0	1.24 a	1.22 a
	250	1.25 a	1.24 a
	500	1.26 a	1.25 a
CWE (mg L^−1^)	0	1.20 c	1.15 c
	5000	1.27 b	1.26 b
	10,000	1.28 a	1.30 a

* Means followed by different small letter(s) within the same column and studied factor, are significantly different according to the LSD test at 0.05 level of probability. CWE: Clove water extract.

**Table 5:** Means of fruit length (cm) as affected by the nitrogen fertilization × potassium silicate and nitrogen fertilizer × clove water extract interaction during 2017 and 2018.

Treatment		Fruit Length					
		Summer 2017			Summer 2018		
Nitrogen fertilization (kg ha^−1^)		144	216	288	144	216	288
Potassium Silicate (mg L^−1^)	0	12.21 * aC	12.96 bB	13.50 aA	13.28 bB	12.91 bC	13.52 bA
	250	12.27 aC	13.36 aB	13.60 aA	13.38 aB	13.38 aB	13.59 bA
	500	12.39 aC	13.44 aB	13.65 aA	13.49 aB	13.47 aB	13.78 aA
CWE (mg L^−1^)	0	11.88 cC	12.99 cB	13.53 aA	12.05 bB	12.98 aA	13.34 aA
	5000	12.31 bC	13.25 bB	13.65 aA	12.34 bB	13.28 aA	13.66 aA
	10,000	12.68 aC	13.52 aB	13.78 aA	15.77 aA	13.50 aA	13.90 aA

* Means followed by different small letter(s) within the same nitrogen fertilization treatment, and different capital letter(s) within the same potassium silicate treatment, for each growing season, are significantly different according to the LSD test at 0.05 level of probability. CWE: Clove water extract.

**Table 6:** Means of fruit total yield (t ha^–1^) as affected by the nitrogen fertilization × potassium silicate during 2017 and 2018.

Season	Nitrogen Fertilization (kg h^−1^)	Potassium Silicate (mg L^−1^)								
		0			250			500		
		Clove Water Extract (mg L^−1^)								
		0	5000	10,000	0	5000	10,000	0	5000	10,000
Summer 2017	144	18.84	20.39	21.24	20.22	22.00	22.05	21.10	22.48	22.43
	216	22.84	23.71	23.95	22.93	23.90	24.36	23.19	24.36	24.84
	288	24.20	25.15	26.22	24.60	25.75	27.06	25.74	28.48	31.71
	LSD_0.05_	1.59								
Summer 2018	144	18.00	21.09	21.36	20.52	21.74	21.88	21.28	22.63	22.29
	216	22.77	23.64	23.88	23.16	23.88	24.43	23.35	24.69	24.91
	288	24.31	25.17	26.54	24.84	25.72	27.21	25.82	28.51	31.22
	LSD_0.05_	0.52								

**Table 7:** Severity of *A. alternata* infection on pepper fruits as affected by the nitrogen fertilization × potassium silicate × clove water extract interaction.

*A. alternata* Rot lesion (mm)	Nitrogen Fertilization (kg h^−1^)	Potassium Silicate (mg L^−1^)								
		0			250			500		
		Clove Water Extract (mg L^−1^)								
		0	5000	10,000	0	5000	10,000	0	5000	10,000
10 DAI *	144	1.50	1.66	1.16	1.50	2.33	1.60	1.50	1.33	1.33
	216	2.66	2.33	2.33	2.56	2.50	2.50	1.33	2.66	1.36
	288	3.00	2.50	2.50	6.83	3.58	3.25	2.83	2.83	1.33
	LSD_0.05_	0.98								
15 DAI	144	2.33	2.66	2.33	5.00	7.00	3.00	7.00	7.50	5.83
	216	2.83	5.75	2.50	9.50	7.83	4.50	2.50	2.90	2.66
	288	5.83	7.75	3.50	8.50	6.50	6.50	5.00	3.60	3.33
	LSD_0.05_	0.93								

* DAI: days after infection.

**Table 8:** Data of average maximum and minimum monthly temperature (°C), and average monthly humidity (%) for the four growing months (February–May) of the two experimental seasons (2017, 2018).

Weather Under Greenhouse Monthly	Seasons	Average Temperature (°C)		Average Humidity %
		Max	Min	
February	2017	19.00	7.78	73.47
	2018	23.55	13.5	75.35
March	2017	23.48	14.63	77.38
	2018	27.58	14.83	78.67
April	2017	27.50	15.80	78.52
	2018	30.37	18.66	79.10
May	2017	32.00	20.83	79.80
	2018	33.49	21.86	79.74

**Table 9:** Physical and chemical properties of the experimental soil of the two growing seasons, 2017 and 2018.

Soil Properties	Season	
	Winter 2017	Winter 2018
Physical		
Sand %	43.3	42.8
Silt %	25.5	23.5
Clay %	31.2	33.7
Soil texture	Sandy loam	Sandy loam
Chemical		
pH	8.45	8.88
E.C.* (dS.m^−1^)	3.01	3.00
Soluble cations (m eq/L)		
Ca^+^	2.43	2.24
Mg^+2^	2.63	2.93
Na^+^	3.59	3.43
K^+^	0.41	0.38
Soluble anions (m eq/L)		
CO_3_^−2^	2.10	2.40
HCO_3_^−^	1.35	1.20
Cl^−^	2.00	1.89
SO^−2^	3.20	3.11
Total N %	0.19	0.15

* E.C.: Electrical Conductivity.

**Table 10:** Schedule of fertigation system applied for nitrogen and phosphorus fertilization of hot pepper plants grown in sandy loam soil under greenhouse in the two growing seasons of 2017 and 2018.

Week after Transplanting	The Rate of N-P (%)	Nitrogen Fertilization			Phosphoric Acid (cm^3^ h^−1^)
		N_1_ (144) kg h^−1^	N_2_ (216) kg h^−1^	N_3_ (288) kg h^−1^	
2	2	2.88	4.32	5.76	3.83
3	4	5.76	8.64	11.52	7.66
4	6	8.64	12.96	17.28	11.49
5	8	11.52	17.28	23.04	15.32
6	12	17.28	25.92	34.56	22.98
7	12	17.28	25.92	34.56	22.98
8	12	17.28	25.92	34.56	22.98
9	12	17.28	25.92	34.56	22.98
10	8	11.52	17.28	23.04	15.32
11	8	11.52	17.28	23.04	15.32
12	8	11.52	17.28	23.04	15.32
13	8	11.52	17.28	23.04	15.32

## Data Availability

Not applicable.
